# Longitudinal relationships between disability and gait characteristics in people with MS

**DOI:** 10.1038/s41598-022-07734-y

**Published:** 2022-03-07

**Authors:** Sapir Dreyer-Alster, Shay Menascu, Mark Dolev, Uri Givon, David Magalashvili, Anat Achiron, Alon Kalron

**Affiliations:** 1 Multiple Sclerosis Center, Sheba Medical Center, Tel-Hashomer, Israel; 2 Sagol School of Neuroscience, Tel-Aviv University, Tel Aviv, Israel; 3 Sagol School of Neurosciences, Tel-Aviv University, Tel Aviv, Israel; 4 Department of Physical Therapy, School of Health Professions, Sackler Faculty of Medicine, Tel-Aviv University, Tel Aviv, Israel

**Keywords:** Signs and symptoms, Disability, Fatigue, Neurology, Multiple sclerosis

## Abstract

Longitudinal data are vital in order to understand intra individual gait changes with the progression of multiple sclerosis (MS). Therefore, the primary aim of this study was to explore the relationship between changes in disability with changes in major spatio-temporal parameters of gait in people with MS (PwMS). PwMS (n = 83) completed two gait assessments performed at separate time points (M1, M2). For each individual, the absolute difference between the Expanded Disability Status Scale (EDSS) score, key spatio-temporal parameters of gait, Falls Efficacy Scale International (FES-I), and the 12-item Multiple Sclerosis Walking Scale (MSWS-12), were calculated. The mean time difference between M1 and M2 was 2.5 (SD = 1.7) years. At M2, PwMS presented with shorter strides, a wider base of support, increased perceived mobility difficulties and fear of falling compared with M1. According to the odds ratio (OR) analysis, the odds of experiencing an increase in the EDSS score was significantly higher once the MSWS-12 score increased at M2 compared with M1 (OR = 7.930, *p* = 0.004). This observation was highlighted specifically in people with mild-moderate MS (OR = 12.427, *p* *<* 0.001). The increase in the EDSS score was not associated with changes in key spatio-temporal parameters of gait. The present study provides a better understanding of gait and disease progression in PwMS, highlighting the significant role of the MSWS-12.

## Introduction

Gait deficiency, one of the main causes of disability in people with multiple sclerosis (PwMS) is one of the most challenging symptoms from the patient’s perspective^1–3^. According to the literature, during the first year post diagnosis, 15% of PwMS use an assistive device for mobility, with 4% requiring bilateral assistance. Furthermore, previous studies have found that 60% of PwMS routinely use at least two mobility devices for ambulation^4^, and use a manual or powered wheelchair or a mobility scooter more frequently compared with the general population^5^. Gait abnormalities in PwMS are not uniform, varying according to location, extent of central nervous system damage, and unpredictability amongst the MS population. Nevertheless, compared with healthy adults, PwMS usually walk at a slower speed, reduced cadence, shorter strides, a prolonged double support phase, increased step time variability and a wider base of support^6^. Moreover, previous studies have found associations between gait abnormalities, an increased risk of falling and fear of falling in PwMS^7,8^.

Walking is a key factor in determining the PwMS’s level of disability. According to the Expanded Disability Status Scale (EDSS), after a full neurological examination, scores ranging up to 4 indicate a fully ambulatory patient; scores between 4.5 and 7.5 indicate mobility restrictions and higher scores denote a worsening condition^9^. Yet, previous studies have found gait abnormalities in minimally and mildly impaired PwMS as well, with EDSS scores ranging < 3^10,11^. Numerous studies have confirmed a positive linear relationship between gait parameters and disability in PwMS^6,12–15^ mainly determining that increased disability is associated with a slower walking speed, reduced cadence, shorter stride length, longer stride time, prolonged double support phase and increased step time variability. However, there is a major limitation with the existing data, as it is almost overwhelmingly based on observational cross-sectional studies, thereby, preventing a clear understanding of the effect of disease progression on the PwMS’s gait pattern.

Filli et al.’s study of 37 PwMS included a baseline and a 1-year follow-up assessment after examining the patient’s gait using a comprehensive 3D gait analysis and clinical walking tests^16^. An increase in knee joint forces was the only change found between assessments, however, a major segment of the sample was lost to follow up. Furthermore, the gait data were collected during treadmill walking, thus, did not completely reflect everyday over ground walking. Hence, there is a consensus amongst the MS scientific community that in order to understand intra individual gait changes with progression of disability, additional longitudinal data are vital.

The primary aim of this study was to explore the relationship between changes in disability (represented by the EDSS scores) with changes in major spatio-temporal parameters of gait in PwMS. We also investigated whether changes in disability and gait were associated with changes in the level of fear of falling.

## Methods

### Study design and participants

Our longitudinal study included 83 PwMS (58.8% females) recruited from the Multiple Sclerosis Center, Sheba Medical Center, Tel-Hashomer, Israel. Data were extracted from the center’s computerized database, a population-based registry documenting demographic, clinical and imaging data of all consecutive PwMS followed at the center. The integrity of the data registry was evaluated by a computerized logic-algorithm-questioning process, identifying data entry errors. A computerized questionnaire helped in choosing PwMS according to the following inclusion criteria: (i) participants were > 18 years old; (ii) no restrictions for MS subtypes; (iii) a neurologist (Neurostatus certified) confirmed diagnosis of definite MS according to the revised McDonald criteria^17^; (iv) disease severity was measured by the EDSS; ≤ 6.5 equivalent to walking ~ 20 m with bilateral support^9^; (v) completion of two computerized gait assessments performed at separate time points (M1, M2) between 1/2012 and 8/2021; and (vi) the computerized gait assessments and a full neurological examination (defining the EDSS score) occurred within a 6 month period. Exclusion criteria included (i) corticosteroid treatment within 60 days prior to gait and/or full neurological assessments; (ii) pregnancy; (iii) other significant neurological illnesses; (iv) cardiovascular, respiratory or orthopedic disorders that could negatively affect mobility; and (v) started or stopped disease modifying treatment within 90 days of gait assessment. All methods were carried out in accordance of relevant guidelines and regulations. Each patient’s record was referenced by an anonymous code to ensure confidentiality during the statistical analyses. The study was approved by the Sheba Institutional Review Board Ethics Committee (Ethics ref. 559608/141210). The approval included waiver of written or verbal informed consent from all study participants. Therefore, individual data will be unavailable to protect the participants’ identity.

### Gait analysis

Temporal-spatial parameters of gait were analyzed by the GAIT Rite system (CIR systems, Havertown, PA, USA), consisting of a 4.6 m long electronic walkway containing 2304 compression-sensitive sensors arranged in a grid pattern. A full description is provided elsewhere^18^. As the subject ambulates across the walkway, pressure is exerted by his feet, thereby, activating the sensors. Simultaneously, targeted software utilizes special algorithms to automatically group the activated sensors and form footprints. The system integrates all footprints and provides the following spatio-temporal parameters: gait velocity, cadence, step/stride length, step/stride time, heel to heel base of support, swing/stance time, single/double time and percentage according to gait cycle. All participants performed six walking trials at a self-selected pace across the electronic walkway. Participants walked with their casual footwear without the use of walking aid/s. A single walking trial was considered valid if the participant walked independently and did not stop when crossing the electronic mat. Gait parameter scores were individually calculated for each pass. The values were subsequently averaged from all the trials to produce the final results. Gait assessment was performed at the Multiple Sclerosis Center, Sheba Medical Center by an experienced physical therapist specialized in neurological rehabilitation.

### Patient reported outcome measures

#### Falls Efficacy Scale International (FES-I)

The participant’s self-report questionnaire, the FES-I^19^, was used to assess the level of concern of falling during 16 activities of daily living ranging from basic to more demanding activities, including social activities that may contribute to the quality of life. Level of concern for each item was scored using a four-point scale (1 = not at all concerned, 4 = very concerned) within a total score range of 16–64; the higher the score, the more the fear of falling. Van Vleit et al. reported that the FES-I is appropriate for research and clinical purposes in PwMS^20^.

#### 12-item Multiple Sclerosis Walking Scale (MSWS-12)

The MSWS-12, a valid questionnaire assessing walking ability in PwMS, is the most widely used patient-reported measure of perceived limitation in walking. Many studies recommend the use of the MSWS-12 due to its psychometric properties^21–23^. Each of the 12 items is rated on a scale of 1 (not at all) to 5 (extremely). Items cover different aspects of walking function and quality, i.e., the ability to walk, walking speed, ability to run, climb up and down stairs, stand, balance, endurance, smoothness of gait, need for support (indoors and outside), effort and concentration required.

#### Statistics

All data were normally distributed according to the Kolmogorov–Smirnov test. Box plots determined outliers for each outcome. For each individual, the absolute difference between the first (M1) and the second measurement (M2) in terms of age, disease duration, EDSS score, walking speed, stride length, stride time, step width, FES-I, and MSWS-12, was calculated. The total cohort was divided into three subgroups; (1) PwMS who demonstrated changes in disability between M1 and M2 within an EDSS range of 0 to 4; (2) PwMS with an EDSS score < 4 at M1 and > 4 at M2; and (3) PwMS who demonstrated changes in disability between M1 and M2 within the EDSS range of 4.5–6.5. The EDSS score of 4.0 was selected as the cutoff for discrimination between subgroups due to its significant relevancy with mobility. PwMS with an EDSS score between 0 and 4 are considered fully ambulatory, denoting walking at least 500 m without a walking aid. EDSS scores ranging between 4.5 and 6.5 differ according to walking distance and type of walking aid^9^. The paired sample t-test determined the differences between M1 and M2 for all outcome measures for the total group and disability subgroups. The Pearson's correlation coefficient examined the associations between the absolute change in disability (EDSS) with the absolute change in walking speed, stride length, stride time, base of support, FES-I, and MSWS-12 for the total group and disability subgroups.

Additionally, subjects were classified as either “Stable EDSS”, i.e. no change in the EDSS score between M1 and M2; or “Increase in EDSS”, i.e. EDSS score at M2 higher than EDSS score at M1 in the total group and disability subgroups. Based on this classification, we calculated the distribution of PwMS with a “worse condition” at M2 compared with M1 for each mobility parameter. A “worse condition” was defined as any decrease in walking speed, shorter stride length, increased stride time, wider step width, additional self-reported mobility difficulties (based on the MSWS-12), and increased fear of falling (based on the FES-I). The OR was calculated separately for each mobility parameter in order to determine the odds of performing worse on a mobility parameter along with an increase in the EDSS score. All analyses were carried out using the SPSS software program (IBM SPSS Statistics for Windows, Version 27.0 Armonk, NY, USA: IBM Corp) and reported p values were two-tailed. The level of significance was set at *p* < 0.05.

## Results

Clinical and demographical characteristics of the total sample and disability subgroups are presented in Table 1. Mean age at M1 was 41.8 (SD = 12.8) years; 86.7% of the PwMS were classified with a relapsing–remitting form of MS. All PwMS included in the sample have been treated with a disease modifying drug for a minimum period of 6 months. The mean time difference between M1 and M2 in the total group was 2.5 (SD = 1.7; range 1.0–6.0) years. The median EDSS score of the total group was 2.0, 3.5 at M1 and M2, respectively. The majority of the cohort (69.9%, n = 58) were PwMS with an EDSS score ≤ 4 at both measurement time points. At M2, PwMS in this subgroup presented shorter strides, a wider base of support, increased perceived mobility difficulties and fear of falling compared with M1. No changes in mobility parameters were observed between the two measurements in PwMS classified with an EDSS score < 4 at M1 and > 4 at M2 (n = 16). PwMS with an EDSS score > 4 at both measurement time points (n = 9) walked slower with shorter strides at M2 compared to M1. Mobility outcome measures according to the two measurement time points in the total group and disability subgroups are presented in Table 1.

**Table 1:** Clinical, demographical characteristics and mobility metrics of the total sample and disability subgroups.

Total group	(*n* = *83*)	M1	M2	Delta M2–M1	*p* value
Gender, F/M		52/31			
Type of MS, RR/P		72/11			
Disease duration (y)		7.0 (8.4)	9.5 (8.9)	2.5 (0.4)	< 0.001
Age (y)		41.8 (12.8)	44.3 (12.8)	2.5 (1.7)	< 0.001
EDSS (median)		2.0 (0–6.5)	3.5 (0–6.5)	1.5	< 0.001
**Mobility parameter**					
Velocity (cm/s)		107.1 (24.9)	101.0 (27.0)	− 5.8 (19.6)	**0.009**
Stride length (cm)		118.4 (18.9)	113.5 (20.6)	− 4.7 (12.4)	**0.001**
Stride time (s)		1.12 (0.14)	1.15 (0.19)	0.03 (0.15)	0.126
Step width (cm)		11.0 (3.3)	11.4 (3.3)	0.34 (2.73)	0.252
MSWS-12 (score)		30.5 (13.9)	34.8 (14.3)	5.2 (13.0)	**0.001**
FES-I (score)		27.8 (10.9)	30.4 (11.4)	3.0 (9.5)	**0.007**

*M1* = *EDSS* ≤ *4* *M2* = *EDSS* ≤ *4*	(*n* = *58*)	M1	M2	Delta M2–M1	*p* value
Gender, F/M		34/24		–	
Type of MS, RR/P		53/5		–	
Disease duration (y)		5.6 (8.7)	9.5 (8.9)	2.5 (0.4)	< 0.001
Age (y)		38.7 (13.5)	41.2 (13.4)	2.5 (1.7)	< 0.001
EDSS (median)		1.5	2.5	1.0 (0.8)	< 0.001
**Mobility parameter**					
Velocity (cm/s)		116.5 (19.9)	112.3 (22.5)	− 4.3 (19.5)	0.128
Stride length (cm)		124.9 (15.3)	121.3 (17.2)	− 3.6 (10.0)	**0.014**
Stride time (s)		1.1 (0.1)	1.1 (0.1)	0.0 (0.1)	0.791
Step width (cm)		10.6 (3.1)	11.5 (3.3)	0.9 (2.5)	**0.015**
MSWS-12 (score)		24.2 (11.4)	30.4 (13.6)	6.2 (12.6)	**0.002**
FES-I (score)		22.8 (6.3)	26.1 (9.5)	3.4 (6.3)	**0.001**

*M1* = *EDSS* ≤ *4* *M2* = *EDSS* > *4*	(*n* = *16*)	M1	M2	Delta M2–M1	*p* value
Gender, F/M		10/6		–	
Type of MS, RR/P		11/4		–	
Disease duration (y)		7.6 (9.5)	10.5 (9.0)	2.9	0.049
Age (y)		45.6 (6.2)	48.5 (7.1)	2.9	< 0.001
EDSS (median)		3.5	5.0	1.5	< 0.001
**Mobility parameter**					
Velocity (cm/s)		84.2 (22.8)	84.8 (22.0)	0.52 (11.9)	0.867
Stride length (cm)		102.1 (21.7)	103.3 (17.3)	1.2 (12.7)	0.730
Stride time (s)		1.2 (0.1)	1.2 (0.2)	0.04 (0.16)	0.319
Step width (cm)		12.0 (3.2)	10.6 (2.5)	− 1.4 (3.3)	0.116
MSWS-12 (score)		44.5 (12.7)	47.5 (8.9)	3.0 (15.6)	0.491
FES-I (score)		39.2 (15.1)	37.3 (8.9)	− 1.9 (14.6)	0.658

*M1* = *EDSS* > *4* *M2* = *EDSS* > *4*	(*n* = *9*)	M1	M2	Delta M2–M1	*p* value
Gender, F/M		8/1			
Type of MS, RR/P		6/2			
Disease duration (y)		11.7 (6.8)	14.4 (5.8)	2.7 (2.7)	0.019
Age (y)		48.8 (9.7)	51.4 (9.5)	2.7 (2.4)	0.011
EDSS (median)		5.0	5.5	0.5 (0.6)	0.053
**Mobility parameter**					
Velocity (cm/s)		91.2 (28.9)	66.1 (24.8)	− 25.1 (19.3)	**0.005**
Stride length (cm)		105.5 (18.4)	86.3 (20.4)	19.2 (11.8)	**0.001**
Stride time (s)		1.2 (0.3)	1.4 (0.4)	0.2 (0.2)	0.080
Step width (cm)		11.4 (3.6)	11.7 (3.9)	0.3 (2.3)	0.715
MSWS-12 (score)		42.0 (9.5)	44.0 (11.8)	2.0 (5.7)	0.327
FES-I (score)		36.5 (9.6)	41.5 (12.0)	5.0 (7.3)	0.094

Significant values are in [bold].

**Figure 1:**
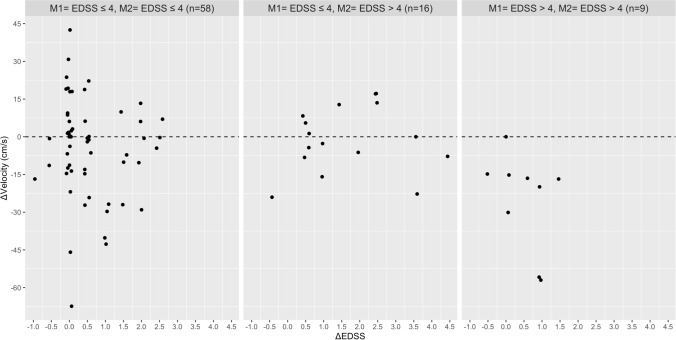
Relationship between the absolute change in walking speed with absolute change in the EDSS score according to disability subgroups.

**Figure 2:**
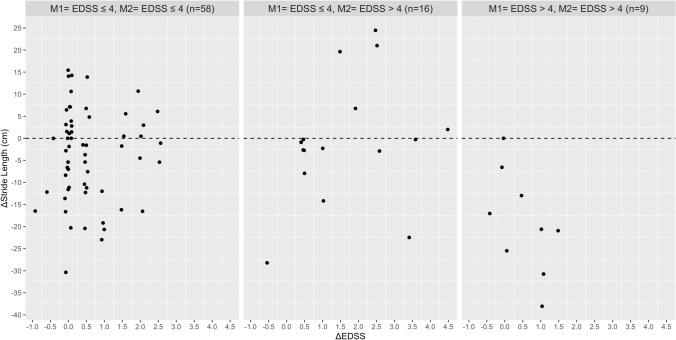
Relationship between the absolute change in stride length with absolute change in the EDSS score according to disability subgroups.

**Figure 3:**
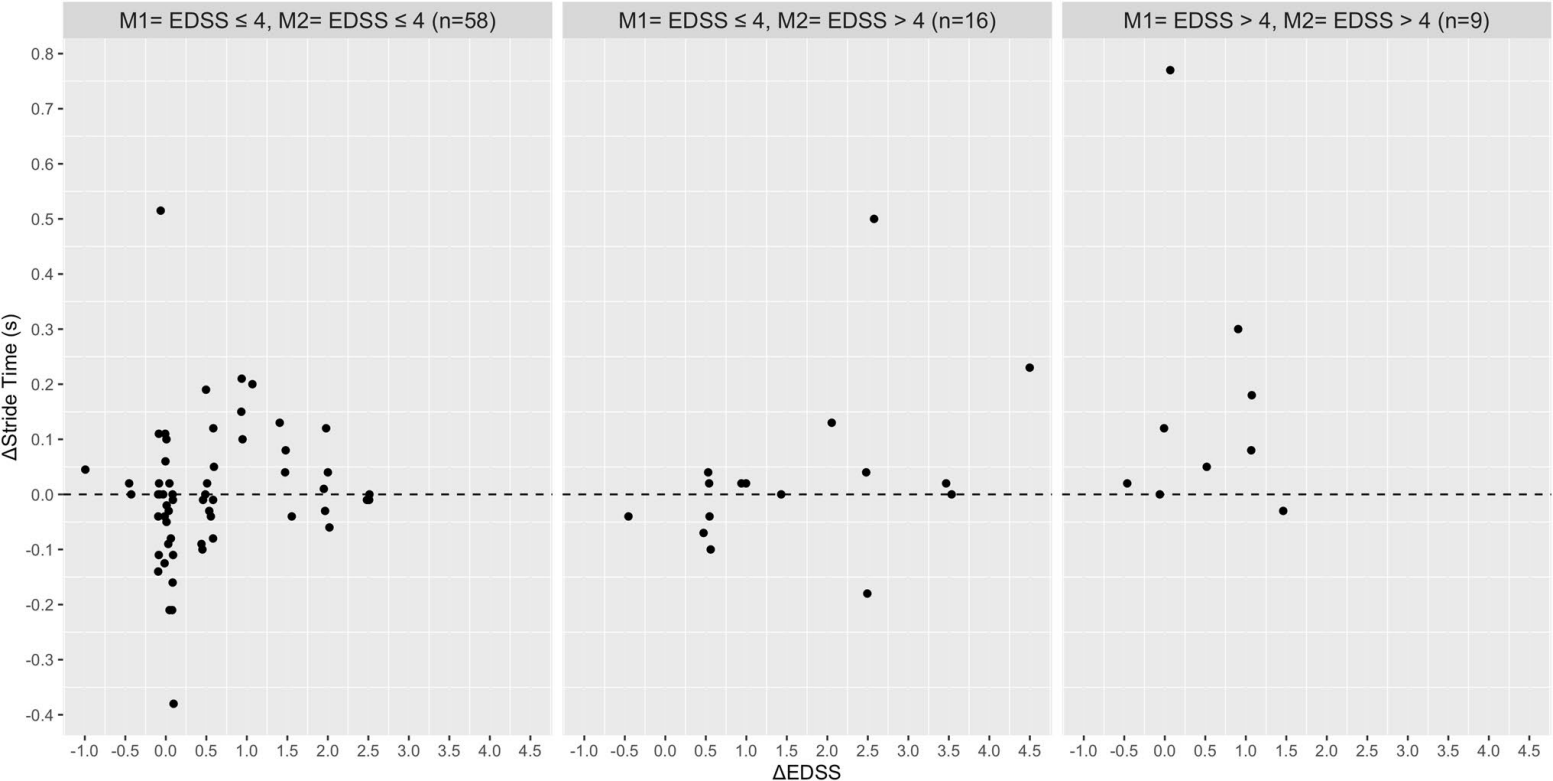
Relationship between the absolute change in stride time with absolute change in the EDSS score according to disability subgroups.

**Figure 4:**
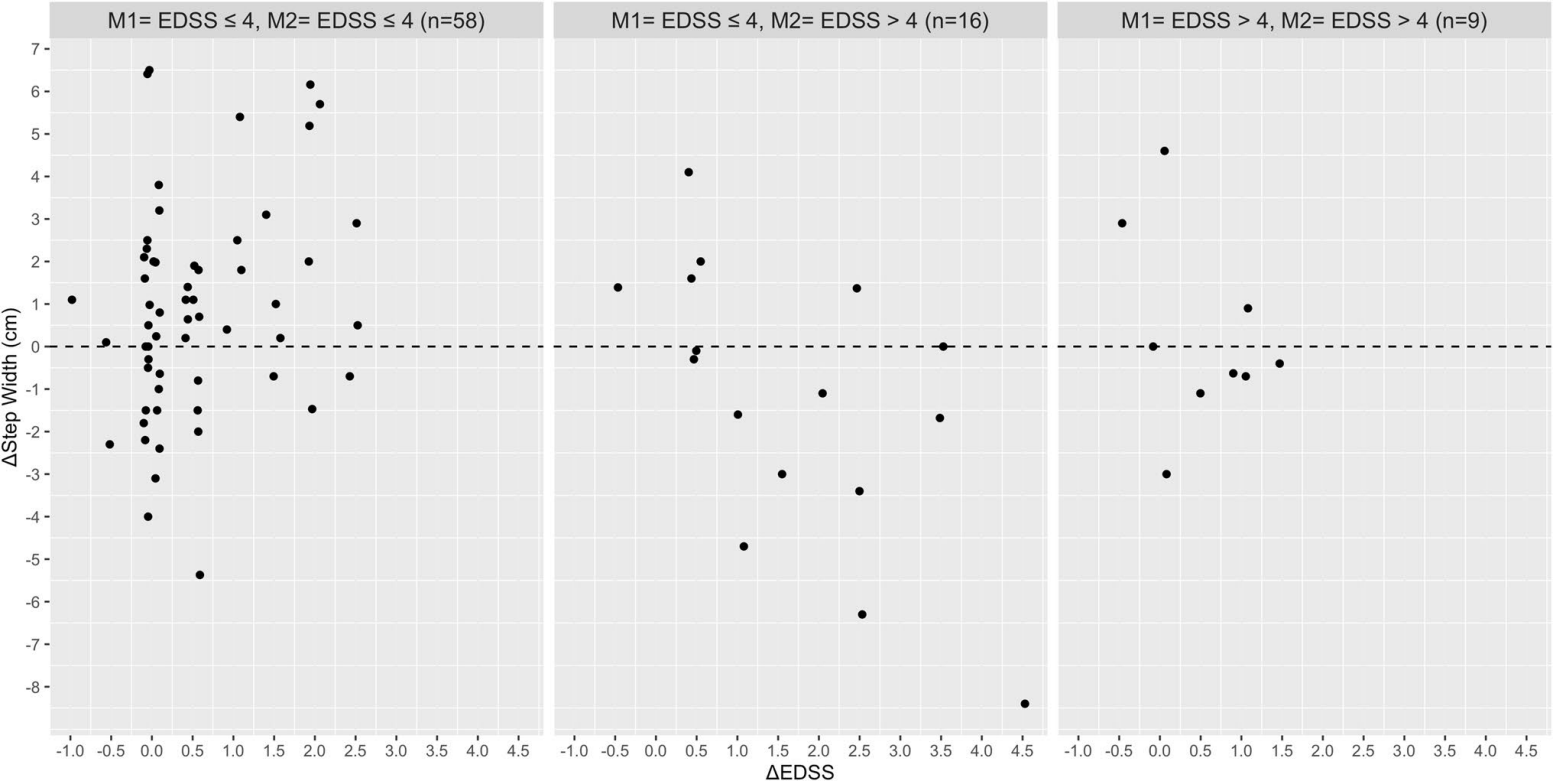
Relationship between the absolute change in step width with absolute change in the EDSS score according to disability subgroups.

**Figure 5:**
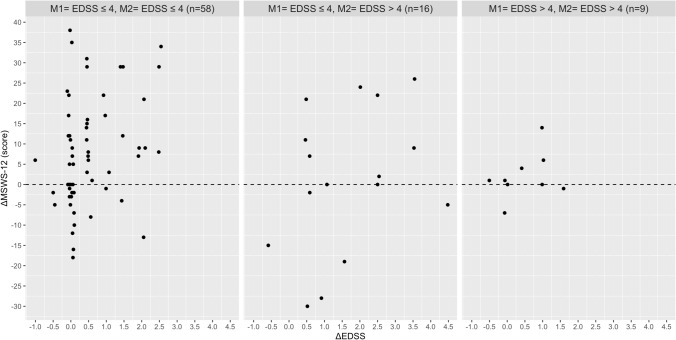
Relationship between the absolute change in the MSWS-12 with absolute change in the EDSS score according to disability subgroups.

**Figure 6:**
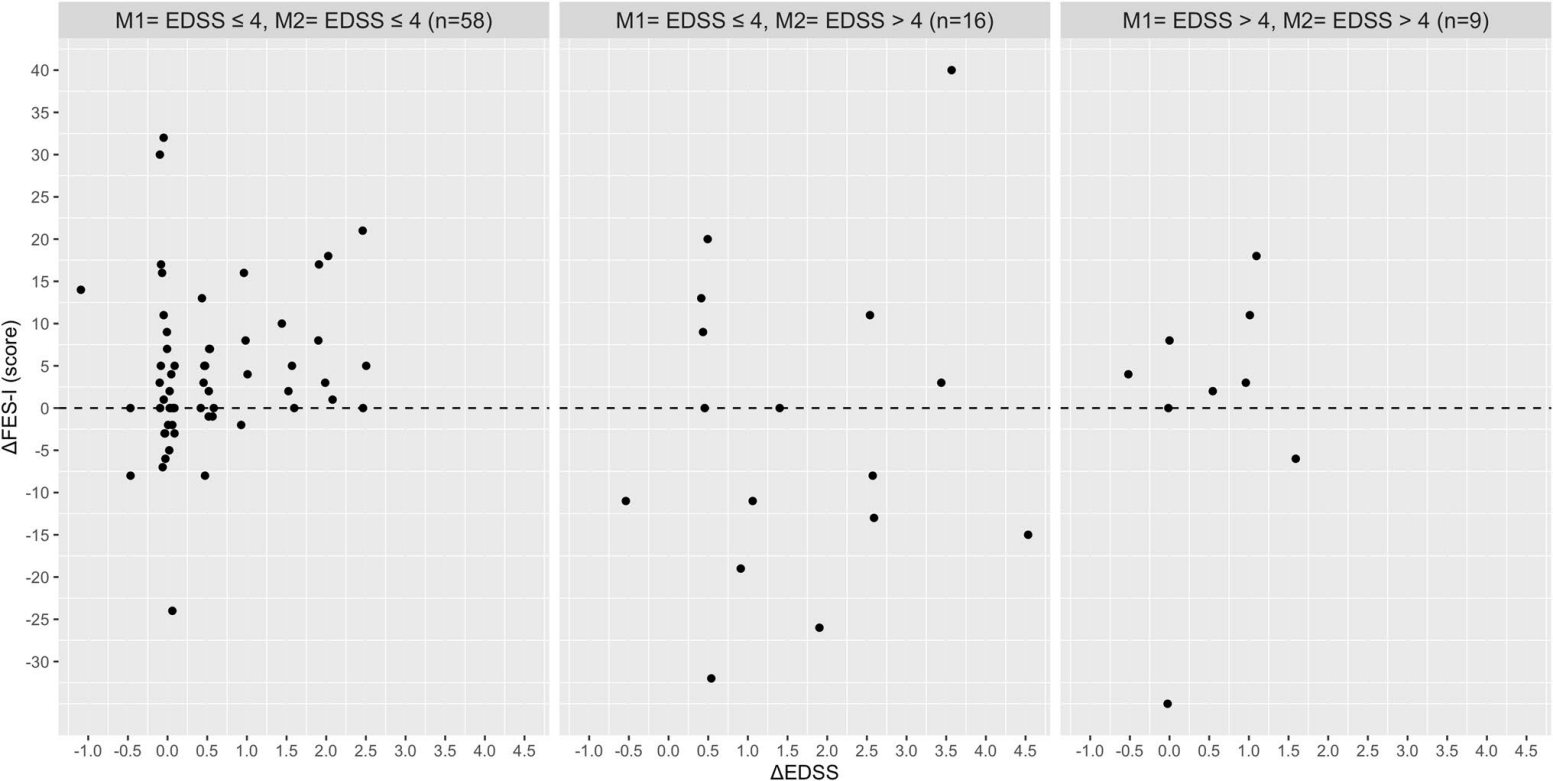
Relationship between the absolute change in the FES-I with absolute change in the EDSS score according to disability subgroups.

Based on the Pearson’s correlation analysis, no relationship was found between the absolute change in EDSS with the absolute change in walking speed, stride length, stride time, MSWS-12 and the FES-I in the total group and disability subgroups (Table 2). In contrast, significant associations were found between the absolute change in the MSWS-12 score with absolute change in walking speed (Rho = 0.444), stride length (Rho = − 0.388), stride time (Rho = 0.436) and base of support (Rho = 0.345) in PwMS with an EDSS score ≤ 4 at M1 and M2. In the same disability subgroup, the increase in fear of falling was correlated with the absolute reduction in walking speed (Rho =  − 0.313), stride length (Rho =  − 0.440), and wider step width (Rho = 0.392). The absolute change in fear of falling (FES-I) and perceived walking difficulties (MSWS-12) were associated with each other in the total group and all three disability subgroups. Figures 1, 2, 3, 4, 5 and 6 illustrate the relationship between the absolute change in gait outcome measures, MSWS-12 and FES-I, with an absolute change in the EDSS score according to disability subgroups.

**Table 2:** Pearson’s correlation coefficient (*p* value) between absolute changes in disability and mobility outcome measures.

*M1* = *EDSS* ≤ *4* *M2* = *EDSS* ≤ *4*	n = 58	Delta EDSS	Delta velocity	Delta stride length	Delta stride time	Delta step width	Delta MSWS-12
Delta velocity		− 0.093 (0.488)	–				
Delta stride length		− 0.019 (0.889)	0.917 **(< 0.001**)	–			
Delta stride time		0.164 (0.219)	− 0.888 (**< 0.001**)	− 0.717 (**< 0.001**)	–		
Delta step width		0.261 (0.148)	− 0.156 (0.241)	− 0.165 (0.217)	0.149 (0.266)	–	
Delta MSWS-12		0.249 (0.069)	− 0.442 (**0.001**)	− 0.388 (**0.004**)	0.436 (**0.001**)	0.345 (**0.011**)	–
Delta FES-I		0.151 (0.275)	− 0.313 (**0.022**)	− 0.440 (**0.001**)	0.071 (0.616)	0.392 (**0.004**)	0.509 (**< 0.001**)

*M1* = *EDSS* ≤ *4* *M2* = *EDSS* > *4*	n = 16						
Delta velocity		− 0.090 (0.750)	–				
Delta stride length		0.102 (0.716)	0.777 (**0.001**)	–			
Delta stride time		0.377 (0.166)	− 0.041 (0.883)	0.417 (0.122)	–		
Delta step width		− 0.573 (0.125)	0.064 (0.821)	− 0.144 (0.609)	− 0.090 (0.751)	–	
Delta MSWS-12		0.092 (0.764)	− 0.293 (0.331)	− 0.300 (0.320)	− 0.002 (0.994)	0.080 (0.796)	–
Delta FES-I		− 0.221 (0.490)	− 0.130 (0.687)	− 0.323 (0.306)	− 0.241 (0.450)	0.354 (0.259)	0.761 (**0.004**)

*M1* = *EDSS* > *4* *M2* = *EDSS* > *4*	n = 9						
Delta velocity		− 0.447 (0.228)	–				
Delta stride length		− 0.524 (0.148)	0.752 (**0.019**)	–			
Delta stride time		− 0.164 (0.674)	− 0.347 (0.360)	0.106 (0.786)	–		
Delta step width		− 0.361 (0.340)	0.185 (0.634)	− 0.355 (0.348)	− 0.462 (0.246)	–	
Delta MSWS-12		0.362 (0.339)	− 0.742 (**0.022**)	− 0.352 (0.353)	0.011 (0.979)	− 0.432 (0.246)	–
Delta FES-I		− 0.014 (0.973)	− 0.837 (**0.009**)	− 0.509 (0.198)	0.365 (0.374)	− 0.065 (0.879)	0.861 (**0.006**)

*Total*	n = 83						
Delta velocity		− 0.038 (0.731)	–				
Delta stride length		0.090 (0.414)	0.873 (**< 0.001**)	–			
Delta stride time		0.173 (0.116)	− 0.657 (**< 0.001**)	− 0.359 (**0.001**)	–		
Delta step width		− 0.150 (0.172)	− 0.116 (0.292)	− 0.206 (0.060)	0.033 (0.764)	–	
Delta MSWS-12		0.169 (0.139)	− 0.331 (**0.003**)	− 0.235 (**0.040**)	0.210 (0.067)	0.249 (**0.029**)	–
Delta FES-I		− 0.055 (0.638)	− 0.311 (**0.007**)	− 0.371 (**0.001**)	0.031 (0.790)	0.373 (**0.001**)	0.596 (**< 0.001**)

Significant values are in [bold].

Table 3 presents the distribution of the total sample according to EDSS status (stable/ increased) and mobility status, i.e. performance at M2 compared with M1 (worse/improved or similar). Fifty-one (out of 83) PwMS presented with a higher EDSS score at M2 compared with M1, whereas, 32 PwMS remained stable (their EDSS score at M2 and M1 was identical). Nineteen, out of the 51 PwMS with an increased EDSS, demonstrated an increase of 0.5 points, 10 participants demonstrated an increase of 1.0 points, and the rest (n = 32) demonstrated an increase > 1.0 point. The maximum increase was 4.5 points. According to the OR analysis, the odds of experiencing an increase in the EDSS score was significantly higher once their MSWS-12 score increased at M2 compared with M1 (OR = 7.930, *p* = 0.004). This observation was highlighted specifically in PwMS who scored < 4 at M1 and M2 (OR = 12.427, *p* < 0.001). OR scores according to disability subgroups are presented in Table 4.

**Table 3:** Distribution according to EDSS status and mobility performance status in the total sample (n = 83).

EDSS status	n (%)	Velocity		Stride length		Stride time		Step width		MSWS-12		FES-I	
		W	I + S	W	I + S	W	I + S	W	I + S	W	I + S	W	I + S
Stable EDSS	32 (44.6%)	18/32 (56.3%)	14/32 (43.7%)	18/32 (56.3%)	14/32 (43.7%)	19/32 (59.4%)	13/32 (40.6%)	19/32 (59.4%)	13/32 (40.6%)	14/32 (43.7%)	18/32 (56.3%)	16/32 (50%)	16/32 (50%)
Increase in EDSS	51 (55.4%)	35/51 (68.6%)	16/51 (31.4%)	36/51 (70.6%)	15/51 (29.4%)	30/51 (58.8%)	21/51 (41.2%)	32/51 (62.7%)	19/51 (37.3%)	38/51 (74.5%)	13/51 (25.5%)	30/51 (58.8%)	21/51 (41.2%)
Odds ratio		1.296 (*p* = 0.255)		1.763 (*p* = 0.184)		0.002 (*p* = 1.00)		0.094 (*p* = 0.759)		7.930 (**0.004**)		0.619 (*p* = 0.432)	

Classification is based on condition at M2 compared with M1. Worsening is considered as any decrease in walking speed, shorter stride length, increased stride time, wider based of support, more self-reported mobility difficulties (MSWS-12), and increased fear of falling (FES-I) at M2 compared with M1.

*W* worsening, *I* improved, *S* similar.

Significant values are in [bold].

**Table 4:** Distribution according to disability subgroups, EDSS status at M2 compared with M1, and mobility performance at M2 compared with M1.

Disability subgroups	EDSS status	n (%)	Velocity		Stride length		Stride time		Step width		MSWS-12		FES-I	
			W	I + S	W	I + S	W	I + S	W	I + S	W	I + S	W	I + S
*M1* = *EDSS* ≤ *4* *M2* = *EDSS* ≤ *4* *(n* = *58)*	Stable EDSS	27 (46.5%)	14/27 (51.9%)	13/27 (48.1%)	14/27 (51.9%)	13/27 (48.1%)	16/27 (59.3%)	11/27 (40.7%)	16/27 (59.3%)	11/27 (40.7%)	12/27 (44.4%)	15/27 (55.6%)	14/27 (51.9%)	13/27 (48.1%)
	Increase in EDSS	31 (53.5%)	21/31 (67.7%)	10/31 (32.3%)	22/31 (71.0%)	9/31 (29.0%)	17/31 (54.8%)	14/31 (45.2%)	24/31 (77.4%)	7/31 (22.6%)	27/31 (87.1%)	4/31 (12.9%)	23/31 (74.2%)	8/31 (25.8%)
Odds ratio			1.526 (*p* = 0.216)		2.248 (*p* = 0.134)		0.115 (0.734)		2.231 (*p* = 0.135)		12.427 (***p* < 0.001**)		3.137 (*p* = 0.077)	
*M1* = *EDSS* ≤ *4* *M2* = *EDSS* > *4* *(n* = *16)*	Stable EDSS	1 (6.3%)	1/1 (100%)	0/1 (0%)	1/1 (100%)	0/1 (0%)	0/1 (0%)	1/1 (100%)	1/1 (100%)	0/1 (0%)	0/1 (0%)	1/1 (100%)	0/1 (0%)	1/1 (100%)
	Increase in EDSS	15 (93.7%)	9/15 (60%)	6/15 (40%)	9/15 (60%)	6/15 (40%)	9/15 (60%)	6/15 (40%)	4/15 (26.6%)	11/15 (73.4%)	8/15 (53.3%)	7/15 (46.7%)	6/15 (40%)	9/15 (60%)
Odds ratio			0.98 (*p* = 0.322)		0.98 (*p* = 0.322)		1.74 (*p* = 0.187)		2.477 (*p* = 0.116)		1.453 (*p* = 0.228)		0.98 (*p* = 0.322)	
*M1* = *EDSS* > *4* *M2* = *EDSS* > *4* *(n* = *9)*	Stable EDSS	4 (44.4%)	3/4 (75%)	1/4 (25%)	3/4 (75%)	1/4 (25%)	3/4 (75%)	1/4 (25%)	2/4 (50%)	2/4 (50%)	2/4 (50%)	2/4 (50%)	2/4 (50%)	2/4 (50%)
	Increase in EDSS	5 (55.6%)	5/5 (100%)	0/5 (0%)	5/5 (100%)	0/5 (0%)	4/5 (80%)	1/5 (20%)	4/5 (80%)	1/5 (20%)	3/5 (60%)	2/5 (40%)	4/5 (80%)	1/5 (20%)
Odds ratio			1.780 (*p* = 182)		1.780 (*p* = 182)		0.032 (*p* = 0.858)		0.908 (*p* = 0.341)		0.09 (*p* = 0.764)		0.908 (*p* = 0.341)	

Classification is based on condition at M2 compared with M1. Worsening is considered as any decrease in walking speed, shorter stride length, increased stride time, wider step width, more self-reported mobility difficulties (MSWS-12), and increased fear of falling (FES-I).

*W* worsening, *I* improved, *S* similar.

Significant values are in [bold].

## Discussion

Our main objective was to examine via longitudinal data whether an increase in the EDSS score, representing worse disability due to MS, is associated with changes in gait characteristics over the same period of time. Our main finding was that the change in the MSWS-12 best reflects changes in the EDSS score over time. Out of the 51 PwMS who presented with an increase in their EDSS, 74.5% (n = 31) also exhibited an increase in the MSWS-12 score over the same time period. Furthermore, out of the 32 PwMS who maintained their EDSS score over time, 56.3% scored similarly on the MSWS-12. Interestingly, these findings were specifically observed in PwMS classified as fully ambulatory (EDSS scores ≤ 4).

Our results reinforce the findings of previous studies reporting on the psychometric measures and responsiveness of the MSWS-12^22,23^. The MSWS-12 is currently the most widely, qualitative, patient-reported outcome measure assessing the patients’ perception of the impact of MS on walking ability^24^. It is frequently employed in clinical trials, particularly, where the interventions are targeted at alleviating walking impairment^25^. According to findings from a large multicenter study coordinated by the European Rehabilitation in MS network (euRIMS), the MSWS-12 demonstrated superior responsiveness in detecting improvements in walking following physical therapy in PwMS compared with various short and long clinical mobility tests^22,23^. Findings were confirmed in both mild and moderate-severe disabled subgroups. Additionally, the clinically meaningful improvement of the MSWS-12 was proposed to be − 8.9 and − 6.3 (0–100 scale) from the patients’ and therapist’s perspective^26^, which corresponds to the value found in the present study, being 5.2 (0–60 scale) in the total group.

We offer a hypothesis of the significant role of the MSWS-12 to capture gait deficits over time in PwMS. We speculate that changes in mobility over time in PwMS comprise two phases, subjective and objective. Symptoms tend to develop over time at different rates, i.e., an attack of symptoms may develop and/or last for several months. In this case, there is a chance that minor difficulties in mobility, specifically, in mildly disabled PwMS, which are difficult to capture during a clinical examination, are better detected by subjective complaints (i.e. captured by the MSWS-12). Only subsequently, when gait difficulties appear, they are eventually captured by an objective measurement tool. Langeskov-Christensen et al. reported that the MSWS-12 captures impairments gradually than the two and six-minute walk test in people with mild MS, thus, suggesting that the MSWS-12 is more sensitive to impairments when evaluating walking in people with mild MS^27^. Worth noting, the MSWS-12 includes questions on balance when standing, and concentration while walking, providing a broader view on mobility difficulties, compared with spatio-temporal parameters of gait. Therefore, it can be assumed that in cases where the main cause of mobility difficulties is poor balance control and/or cognitive impairment, the MSWS-12 score better captures these symptoms compared with measures of standard spatio-temporal parameters of gait.

Interestingly, the increase in the EDSS score was not associated with a decrease in walking speed, shorter strides, prolonged stride time, wider step width or increased fear of falling. We assume that the main reason is partially due to the range of disability of the present cohort. For the majority of the participants included in this analysis, the increase in the global EDSS score occurred within the 0 to 4 range. Changes within this range are mainly based on the worsening of the independent functional systems. There is a possibility that for several PwMS, the increase in the global EDSS score was due to a worsening in functional systems which less likely effected mobility, i.e., visual and/or bowel and bladder and/or cerebral. This assumption might also explain the large variance of gait performance, despite an increase in the EDSS score. Nevertheless, it is worth mentioning that previous studies have found significant differences in spatio-temporal parameters of gait within the lower range of the EDSS score indicating minimally-mildly impaired PwMS^10,24^. Preiningerova et al. reported that walking speed and step length significantly differs between PwMS with EDSS scores of 2.0–2.5, 3.0–3.5 and 4.0–4.5^28^. Therefore, in order to further clarify this issue, we encourage future research to examine the relationship between changes in disability and gait over time in PwMS by differentiating disability according to the functional systems. Furthermore, it would be interesting to add other measures of gait, such as variability of strides, as they may more accurately capture changes in disability compared with the major metrics of gait^13^.

This study is not without limitations. Firstly, the difference between M1 and M2 was defined according to the EDSS score, not by a predefined period of time, thereby, denoting that the time frame between measurements was different between the PwMS. Accordingly, it may be argued that age might have served as a confounding factor. Nevertheless, we claim that key gait characteristics such as speed and step length are stable during adulthood up to the seventh decade of life. Notably, when selecting a predefined time period between measurements, it is possible that the rate of disease progression varies between PwMS. In the same context, the neurological examination (determining the EDSS) and gait assessment at each time point (M1/M2) were not performed on the same occasion. Nevertheless, we confirm that the participants did not experience a neurological attack (due to MS) and/or a significant physical deterioration in between the EDSS and gait assessments at each time point. Secondly, our analysis did not include other factors related to mobility, such as spasticity, postural control, muscle strength and endurance of the lower limbs. Furthermore, our analysis did not differentiate between types of MS, mainly due to the limited amount of PwMS with a progressive form of the disease. Therefore, future research is encouraged to explore whether our findings are accurate in PwMS with a progressive form, characterizing a different rate of disease progression compared with the relapsing–remitting form. Finally, the effect of different immunomodulatory drugs on definite spatio-temporal parameters of gait in PwMS is currently unknown.

## Conclusions

The present study provides a better understanding of gait and disease progression in PwMS. Health professionals should be aware that despite maintaining a stable EDSS score over time, many PwMS experience deficits in key walking parameters during the same period. Moreover, an increase in the MSWS-12 appear to accurately indicate an increase in disability. New longitudinal studies should be conducted to include additional elements of mobility (i.e., postural control, muscle strength and endurance) in order to fully capture changes in the gait pattern in vis a vis disability and MS progression.
